# Insurance Status, Language, and Access to Care for Pediatric Anterior Cruciate Ligament Injury

**DOI:** 10.1177/23259671241270310

**Published:** 2024-09-04

**Authors:** Anna L. Park, Pardeep Singh Dhillon, Nirav K. Pandya

**Affiliations:** † School of Medicine, University of California, San Francisco, San Francisco, California, USA; ‡ Department of Pediatric Orthopaedic Surgery, University of California, San Francisco, Benioff Children's Hospital Oakland, Oakland, California, USA; Investigation performed at University of California, San Francisco, School of Medicine, San Francisco, California, USA

**Keywords:** ACL, disparity, insurance, socioeconomic, pediatric sports medicine, language

## Abstract

**Background:**

Patients with public insurance (PUBs) face more difficulty obtaining orthopaedic appointments and have longer wait times than privately insured patients (PVTs). These delays are associated with greater injury severity at the time of surgery, which affects sports injuries such as anterior cruciate ligament tears where early surgical stabilization leads to better outcomes. Additionally, previous evidence showed that patients with limited English proficiency often must rely on informal translation services, such as family members or friends, to communicate with their orthopaedic surgeons, which may represent a disparity in the care provided.

**Hypothesis:**

It was hypothesized that PUBs would be less likely to obtain an appointment compared with PVTs and that most providers would not offer professional translation services to Spanish-speaking patients.

**Study Design:**

Cross-sectional study.

**Methods:**

The authors called 50 randomly selected orthopaedic surgeons’ offices in California specializing in sports medicine to request an appointment. Each office was called 4 times in random order for the hypothetical patient having either private or public insurance and speaking either Spanish or English.

**Results:**

The hypothetical PUB had significantly decreased access to an appointment (19% of offices offered an appointment) when compared to the PVT (73.8% offered an appointment). Independent private practice (IPP) offices were less likely to accept public insurance (13.3%) compared with offices at academic medical centers (57.1%). There was no difference in access to an appointment for the Spanish- versus English-speaking patient. Translation services were offered at 73.8% of the orthopaedic offices.

**Conclusion:**

Overall, the data illustrated disparities in access to pediatric orthopaedic care for PUBs compared to those with private insurance. Disparities were most prominent in IPP settings, which were less likely than academic offices to accept public insurance. Additionally, it was found that 73.8% of the offices the authors contacted offered Spanish translation services. Interventions should focus on increasing acceptance of public insurance and translation services in IPP settings. Future studies should expand this analysis to other languages and investigate the potential impacts of language on the quality of care provided.

Medicaid and the Children's Health Insurance Program are the largest insurance providers for children in the United States. These public programs collectively enrolled >39 million children as of May 2021.^5,19^ While the number of children with health insurance across the United States has increased, it has not necessarily translated into improved access to care. Research on access to orthopaedic care has demonstrated that patients with public insurance (PUBs) are less likely to secure appointments^13^ and have longer wait times than privately insured patients (PVTs).^3^ Such delays are correlated with more severe injury at the time of surgery^15,17,23^ and are particularly relevant for sports injuries such as anterior cruciate ligament (ACL) tears, for which early surgical stabilization has been shown to decrease the risk of chondral injuries and meniscal tear, while also improving knee stability and increasing the ability to return to sports.^17,24^ Delayed or nonoperative treatment, by contrast, has been shown to correlate with increased risk of secondary cartilage damage associated with long-term degenerative consequences.^8^ ACL tears are increasing in frequency as early intensive sports training increases,^16,21^ with the total number of pediatric ACL reconstructions increasing nearly 3-fold relative to all pediatric orthopaedic surgeries in recent decades^27^; therefore, it is important to understand the barriers and disparities in accessing orthopaedic care for pediatric patients presenting with ACL injuries.

Communication barriers also impede the provision of optimal care.^29^ Over 20% of the US population >5 years of age speaks a language other than English at home, the majority of whom speak Spanish, and >8% of the population has limited English proficiency.^28^ Recent evidence suggests that there is no disparity between Spanish-speaking and English-speaking patients’ access to orthopaedic appointments; however, patients with limited English proficiency often have to rely on family members or nonprofessional services to communicate with their physicians, representing a disparity in the quality of care they receive.^11^

Access to care may also vary by practice setting. Academic medical centers (AMCs) are often affiliated with universities and provide patient care while also educating health care providers. Surgeons may also have their own independent private practices (IPPs), which must negotiate contracts for reimbursement individually with insurance companies. Finally, Kaiser is a type of managed care consortium that provides insurance to patients in a health maintenance organization (HMO) model. In this model, patients are limited to seeing doctors working within the organization. Given the evolving nature of insurance coverage and care access, updated analysis of care access is necessary for understanding disparities in orthopaedic care.

The purpose of this study was to provide updated data on how insurance status affects the ability of pediatric patients with an ACL tear to get an appointment and to analyze how these disparities vary by type of practice. Additionally, this study addressed the potential intersectionality of care disparities by combining analysis of language barriers with the analysis of insurance status. Based on the existing literature, we hypothesized that PUBs would be less likely to obtain an appointment than PVTs and that most providers would not offer professional translation services to Spanish-speaking patients. Furthermore, we also hypothesized that providers affiliated with AMCs would be more likely to accept public insurance and offer translation services than providers working in IPP.

## Methods

### Participants

Using the online “Find an Orthopaedist” search tool provided for patients by the American Academy of Orthopaedic Surgeons (AAOS), we generated a list in August 2023 of all 414 orthopaedic surgeons in California with an identified specialty in “sports medicine.” A random number generator was then used to select the offices of 50 orthopaedic surgeons to include in this study.

### Procedures

**Appendix Figure A1.**
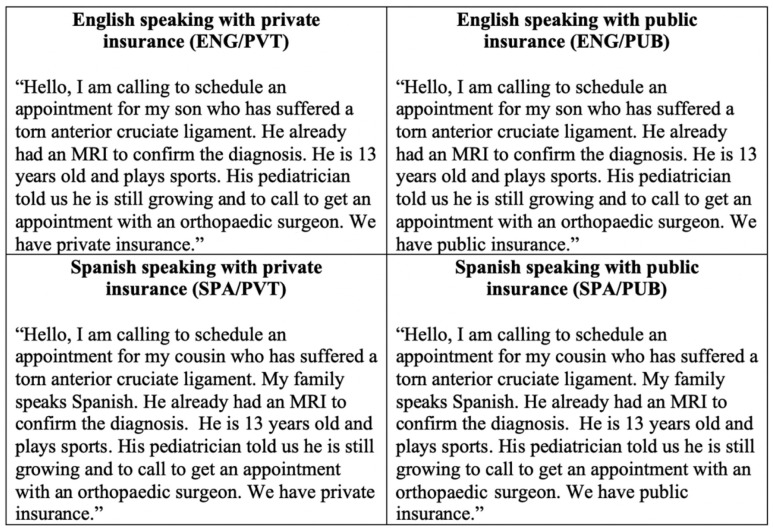
Telephone scripts. MRI, magnetic resonance imaging.

Study design including procedure and script development was led by a pediatric orthopaedic surgeon (N.K.P.) with specialization in sports medicine and experience working with PUBs. The investigators called each of the 50 randomly selected offices to request an appointment for a relative with an ACL tear already confirmed on magnetic resonance imaging. Each of the offices were called 4 separate times, each time with 1 of 4 different scripts, in random order (Appendix Figure A1). For each call, the following data were collected: (1) whether the patient was offered an appointment; (2) whether the patient was given an appointment time; (3) whether the patient's citizenship status was asked; (4) whether the provider was a pediatric specialist; and (5) whether the provider had access to interpretation services for the Spanish-speaking patient. All telephone calls were conducted in English. Calls and data collection were performed by a medical student (P.S.D.). Due to the nature of the study, informed consent was not able to be obtained from the orthopaedic offices; however, ethical approval was obtained through our institutional review board (UCSF IRB No. 23-39135).

### Statistical Analysis

Analyses were performed by 2 authors (A.L.P. and P.S.D.) with direction from the senior author (N.K.P.). Orthopaedic offices were classified by type of practice: AMC, IPP, and Kaiser NorCal (Kaiser). Descriptive statistics were used to summarize the data. Odds ratios were calculated using Prism 10 (GraphPad), and confidence intervals were determined using the Baptista-Pike method with an alpha level of 0.05.

## Results

### Provider Characteristics

Of the 50 orthopaedic offices called, 1 office was closed becasue the provider had retired. From the remaining 49 orthopaedic providers, 7 were AMCs, 37 were IPPs, and 5 were Kaiser. Seven of the IPPs were not able to be reached by telephone and were excluded from data.

### Access to an Appointment

A total of 34 (73.8%) orthopaedic offices contacted offered the PVT an appointment, while only 8 offices (19%) offered an appointment to the PUB (Table 1). The odds ratio for not being offered an appointment when calling an orthopaedic office for the PUBs relative to PVTs was 3.09 with a 95% CI of 1.38 to 6.90. Language spoken (English or Spanish) did not affect whether the patient was offered an appointment. All of the 8 offices offering the PUB an appointment required supplemental insurance and a referral from a primary care provider. The 1 office accepting public insurance without these requirements did not provide an appointment time.

**Table 1:** Contingency of Offices Offering Appointments^*a*^

	No Appointment	Appointment	Total
PUB	34	8	42
PVT	11	31	42
Total	45	39	84

a Data are presented as n. PUB, publicly insured patient; PVT, privately insured patient.

**Figure 1.**
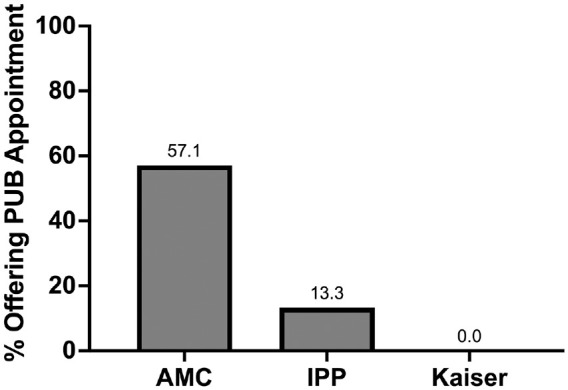
Offices offering appointments for publicly insured patients (PUBs). AMC, academic medical center; IPP, independent private practice; Kaiser, Kaiser NorCal.

Four of the providers accepting public insurance were AMCs and 4 were IPPs. Given the distribution of providers in the sample, 57% of AMCs accepted public insurance, and 13.3% of IPPs accepted public insurance. Kaiser, as an HMO, does not accept public insurance (Figure 1).

Some offices said they could offer an appointment but did not offer an actual appointment time for every patient. One provider (IPP) offered appointment times to the English-speaking PVT but required further insurance information details (policy number) and did not offer appointment times for the Spanish-speaking PVT. Two providers (IPPs) required repeat imaging before offering an appointment to the English-speaking PVT but did not require repeat imaging for the Spanish-speaking counterpart. No differences were observed between English-speaking and Spanish-speaking PUBs with respect to appointment offers (Table 2).

**Table 2:** Breakdown of Providers Offering Appointment Times^*a*^

	AMC	IPP	Kaiser	Total
Total providers	7	30	5	42
Accepting PUB	4	4	0	8
English-speaking	4	4	0	8
Spanish-speaking	4	4	0	8

a Data are presented as n. All offices required supplemental insurance and primary care provider referral to schedule an appointment. AMC, academic medical center; IPP, independent private practice; Kaiser, Kaiser NorCal; PUB, publicly insured patient.

### Interpreter Services

**Figure 2.**
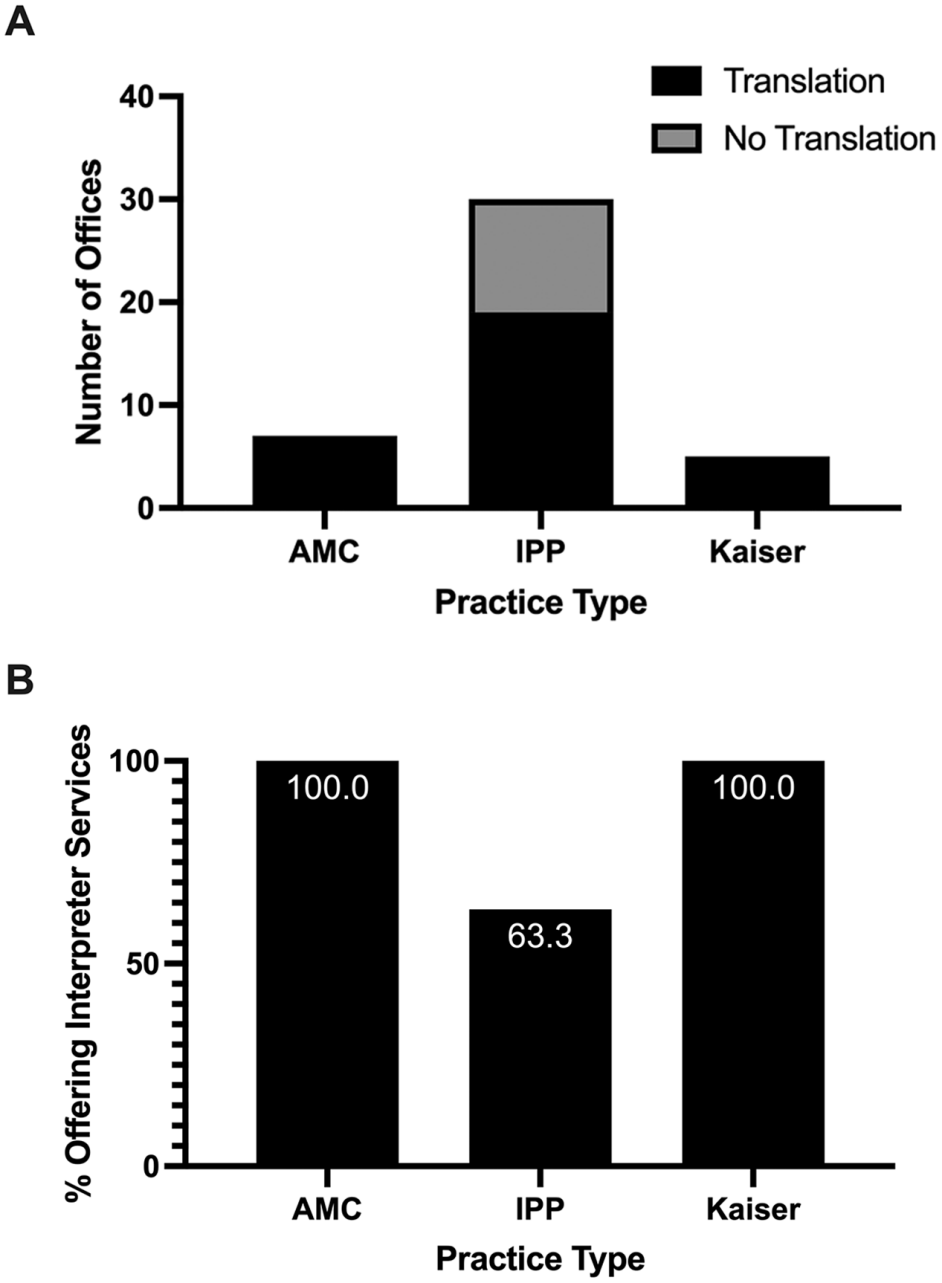
Providers offering professional interpreter services for the Spanish-speaking patient. (A) Total number of offices offering translation services by practice type. (B) Percentage of offices offering translation services by practice type. AMC, academic medical center; IPP, independent private practice; Kaiser, Kaiser NorCal.

In total, 73.8% of the providers offered interpreter services for the Spanish-speaking patient, with no difference by insurance type. Note that 2 IPPs offered translator services only with workers’ compensation but are not included in our analysis as offering translation services because workers’ compensation is less likely to apply to the pediatric population presenting with a sports-related ACL injury. When further broken down by type of practice, all AMC and Kaiser practices offered interpreter services, while 19 of the IPPs (63.3%) offered translation services (Figure 2). Note that the office that offered appointment times to the English-speaking but not Spanish-speaking patient offered translation services.

### Length to Appointment

English-speaking PVTs were offered appointment times at a mean of 9.88 days out from the time of the call (median, 2.5 days; SD, 12.35 days). On the other hand, Spanish-speaking PVTs were offered appointment times at a mean of 7.71 days (median, 5 days; SD, 7.89) (2-tailed independent *t* test; *P* = .55). Of note, not every office that offered an appointment was able to provide a specific date of appointment. Of the 17 offices that provided the Spanish-speaking patient an appointment date, 64.7% were offices with interpreter services offered.

### Citizenship Status and Pediatric Specialists

No offices inquired about the patient's citizenship status. Nine (21.4%) of the offices had the option for providers that were pediatric specialists.

## Discussion

The major findings of our study demonstrate decreased access to orthopaedic care for pediatric PUBs with ACL tears in northern California when compared with PVTs. Of the total orthopaedic offices contacted, 73.8% offered an appointment to the PVT, whereas only 19% of the offices offered the PUB an appointment. This significant barrier to accessing orthopaedic care for pediatric PUBs compared with PVTs is consistent with data presented in Pierce et al^22^ showing decreased access to orthopaedic care for children with public insurance in the Greater Cincinnati area. Considering that roughly half of the US pediatric population has public insurance, this barrier to access suggests that many patients are unable to access care from the majority of orthopaedic providers.

In addition to the difficulty obtaining an appointment, the 8 offices that accepted public insurance required supplemental insurance and a primary care provider referral to schedule the appointment. This creates additional financial barriers to access to care. Insurance plans vary in their coverage of medical interventions—including surgery. Lack of transparency in the price of procedures, complex insurance payment structures including “deductibles” and “out of pocket maximums,” and the necessity of multiple appointments (eg, primary care provider appointment for referral) make understanding the financial burden of ACL surgery difficult. Patients without insurance, or with inadequate insurance, are left to pay surgery fees out of pocket. For an ACL surgery, a procedure with a national average cost of around $29,590, these bills are often prohibitively high.^18^

Additional barriers for pediatric PUBs are directly related to established socioeconomic, racial, and ethnic disparities within the American health care system. PUBs are more likely to be from lower socioeconomic status and racial and ethnic minority groups. The recent National Health Statistics Report on demographic variation in health insurance coverage showed 87.4% of people with a family income below the federal poverty line had public insurance, and 7.7% were uninsured. By contrast, for families with income >400% of the federal poverty line, 8.0% had public insurance, only 1.9% were uninsured, and 91.1% had private insurance.^6^ In addition to the socioeconomic inequities, racial and ethnic differences are also evident between Americans with private and public insurance. As of 2020, 74.6% of non-Hispanic White and non-Hispanic Asian individuals had private insurance compared with 48.6% of non-Hispanic Black and 44.9% of Hispanic people. Conversely, public coverage was highest among non-Hispanic Black (42.6%), non-Hispanic other/multiple races (38.6%), and Hispanic (34.3%) individuals.^6^ Given these background conditions of the American health insurance system, decreased access to orthopaedic care for PUBs when compared with PVTs is also a matter of racial and ethnic equity.

Within musculoskeletal care, racial and ethnic disparities have been shown in minorities’ lower utilization rates for total joint arthroplasty,^2,20^ higher rates of complication and readmission after orthopaedic procedures,^7,9^ and increased morbidity and mortality after hip fracture.^9^ The reasons behind these disparities are complex and multifactorial; however, our findings suggest that increasing access to orthopaedic care for PUBs may improve health equity in line with national initiatives^12^ aimed at reducing health disparities.

Further analysis of our data revealed differences in access by practice type. In our sample, there were >4 times more IPP providers when compared with AMCs (30 IPP and 7 AMC in the random sample), a distribution congruent with the national 14% of providers working in academic practice as reported in the 2019 AAOS report on orthopaedic practice in the United States.^1^ In our sample, 57% of AMCs offered the PUB an appointment, compared with only 13% of IPPs. Because most orthopaedic providers nationally work in IPP settings, our findings indicate that reducing disparities in access to orthopaedic care needs to focus on IPP settings. Kaiser, as an HMO, does not accept public insurance. However, Kaiser is a large provider of insurance in northern California through Medi-Cal, covering >718,000 patients with Medicaid and the Children's Health Insurance Program, of which 46.7% are children.^14^ Through its participation in Medi-Cal, Kaiser presents a unique insurance option that is a blend of private and public insurance.

Previous data on how practice type affects access to care are mixed. Shi et al^25^ reported no difference in access to care by practice type nationally using a similar call-based sampling method for a “patient” with an ACL tear; however, they aggregated data across states, with calls to 4 different practices in each state. Conversely, another study conducted in Illinois suggests that in some regions, AMCs may be more likely to offer specialty care to low-income pediatric PUBs.^3^ Given that public insurance programs differ by state and that geography influences the distribution of orthopaedic services,^10,26^ further region-specific analysis would be necessary to determine whether the findings we observed in northern California are replicated in other geographic regions.

In addition to insurance status, our study also investigated how primary language spoken may affect access to quality care. More than 20% of the US population aged >5 years speaks a language other than English at home, of which Spanish is most common.^28^ Fitting with recent data, we found no significant difference in access to an appointment for Spanish- and English-speaking patients.^11^ Two providers required additional imaging from the English-speaking PUB but did not for the Spanish-speaking counterpart. This could have many explanations, potentially including insurance reimbursement protocols or lack of clear communication between the medical office and the patient.

While access to an appointment is important, appropriate translation services are also crucial to providing adequate quality care.^29^ Analysis of the translation services offered shows that interpreter services were equally available for the PVT and the PUB. In total, 73.8% of providers offered professional translation services. This is comparable with data reported in Greene et al,^11^ in which 80% of patients were asked to bring a friend or family member to translate for the patient. While our findings suggest continued disparity in access to care for Spanish-speaking patients, further analysis is necessary to understand if quality of care is comparable for Spanish- and English-speaking patients even with translation services.

Analysis of translation services by practice type revealed that 100% of both Kaiser offices and AMCs offered translation services, compared with 63.3% of IPPs. This indicates that strategies for increasing the availability of translation services should target independent providers—perhaps by making such services easier to implement in outpatient clinics, or by incentivizing its incorporation into private practices. Such incentivization might include policies around insurance reimbursement for translation services given that translation services are covered by a patient's insurance in the United States.

Our data also illustrated that the amount of time to an appointment was not statistically significantly different for the English-speaking or Spanish-speaking patient. For ACL surgeries specifically, this is important given that early surgical stabilization has been associated with decreased risk of chondral or meniscal injuries.

Finally, of all the orthopaedic sports medicine offices called, 9 had an option for a surgeon who is a pediatric specialist. Pediatric patients may have different priorities or needs from adult patients, such as student athlete–specific mental health support.^30^ Additionally, pediatric ACL reconstruction requires understanding of skeletal maturity and physeal anatomy to inform best practices.^21^ Given that sports-related ACL injuries are increasingly common in pediatric patients, it is important that surgeons operating on pediatric patients be aware of the unique challenges and concerns relevant to pediatric patients.

### Limitations

There are several limitations to our study. First, it focused on a single geographic region with a large Spanish-speaking population, limiting the generalizability to other geographic regions. Second, our study focused on initial access to an appointment. Other factors may affect ability to arrive at the appointment (eg, transportation, flexible work or school schedule) and there may be other disparities related to quality of care (eg, wait times between appointments, access to physical therapy postsurgery). Third, our data do not indicate why some providers may not accept public insurance or offer translation services. Future studies should investigate the root causes to determine if the barriers are financial (eg, reimbursement rates), logistical (eg, finding certified translators), or for other reasons. Such information would be instrumental in designing efforts to improve access and equity within orthopaedic care. Last, many languages other than English and Spanish are spoken in the United States, and future analysis should examine access for patients who speak languages such as Chinese (Cantonese or Mandarin), Vietnamese, or Tagalog—the next most common languages in California.^4^

## Conclusion

Our data illustrated disparities in access to pediatric orthopaedic care for PUBs compared to those with private insurance. Disparities were most prominent in IPP settings, which were less likely than academic offices to accept public insurance. Additionally, we found that 73.8% of the offices we called offered Spanish translation services. Interventions should focus on increasing acceptance of public insurance and translation services in IPP settings. Future studies should expand this analysis to other languages and investigate the potential impacts of language on the quality of care provided.
